# Removing anonymity protection and utilization review decisions: a real-world case under a single-payer health system

**DOI:** 10.1038/s41598-022-16536-1

**Published:** 2022-07-16

**Authors:** Chih-Kuang Wang, Shih-Jung Chien, Po-Chang Lee, Shou-Hsia Cheng

**Affiliations:** 1 Department of Planning, National Health Insurance Administration, Taipei, Taiwan; 2 Department of Medical Affairs, National Health Insurance Administration, Taipei, Taiwan; 3 National Health Insurance Administration, Taipei, Taiwan; 4 Institute of Health Policy and Management, National Taiwan University, 17 Xu-Zhou Road, Room 618, Taipei, 10055 Taiwan; 5 Population Health Research Center, National Taiwan University, Taipei, Taiwan

**Keywords:** Health policy, Health services, Health care

## Abstract

The effects of anonymity on utilization review has never been examined in the real world. This study aimed to evaluate the impact of removing anonymity protection for claims reviewers on their review decisions. Using a single-blinded repeated measures design, we randomly selected 1457 claims cases (with 12,237 orders) that had been anonymously reviewed and reimbursed in 2016 and had them re-reviewed in a signed review program in 2017 under the Taiwanese National Health Insurance scheme. The signed review policy significantly decreased the likelihood of a deduction decision at the case and the order level (*P* < 0.001). Furthermore, signed reviewers tended to make more “too lenient” decisions, and were less likely to make “too harsh” decisions. Removing anonymity protection dramatically reduced the deduction rate and overturned the tendency of decisions from “too harsh” to “too lenient”. However, whether to maintain the anonymity of utilization reviews is a challenge for health authorities around the globe.

## Introduction

Utilization review has been a routine practice for health care services under public or private insurance schemes. The purpose of utilization review or claims review in the reimbursement process is mainly to contain the unnecessary use of diagnostic and treatment services as well as to ensure the appropriateness and quality of health care^1,2^. In the United States, Germany and Canada, some professional (peer) review organizations provide services for health insurers to conduct claims reviews^3–5^. On the other hand, the single payers in Taiwan and South Korea perform the claims reviews by themselves with the assistance of contracted utilization reviewers^6,7^. Although the quality of utilization review has been challenging for decades^1,2,8–10^, claims review commonly exists in health insurance schemes at present.

Under the universal and compulsory National Health Insurance (NHI) scheme in Taiwan, more than 90% of the clinics and hospitals are contracted with the NHI. Contracted providers have to file claims to the NHI Administration for reimbursement every month electronically, with diagnoses and detailed records of all medical orders provided for every case they served. The NHI Administration conduct professional utilization reviews by contracted claims reviewers to decide the amount of reimbursement for each provider. Usually, 2–5% of the filed cases submitted by contracted providers will be randomly selected for utilization review. For example, an inpatient case with 60 diagnostic and therapeutic orders (items) is filed for reimbursement with a total amount of $30,000 and it is randomly selected for utilization review. The claims reviewer will review the orders and determine the reasonability for each of them based on clinical guidelines and NHI regulations. Once the reviewer consider that certain orders are “not medically necessary “, the amount of money incurred by those orders would be deducted from the claimed reimbursement.

Anonymous review has been popular in professional practices such as academic publication or the institutional review board (IRB). The design of anonymous review is intended to reduce potential reprisals by authors^11^. The pros and cons of open peer review have been discussed in the academic publishing process for many years^12–15^. A recent study by Bravo and colleagues revealed that open peer review does not compromise the review process; however, only a small proportion of reviewers are willing to disclose their identity^16^.

Regarding health care utilization review, which may reduce reimbursement significantly, there is still debate about the anonymity of insurance claims reviewers as a form of protection. Peer Review Organizations (PROs) in the US perform insurance claims review with anonymous protection, as do reviewers in South Korea and Taiwan. In order to pursue transparency in the review process and in response to the request from medical societies in Taiwan, the single payer of the universal health insurance in Taiwan (i.e., the National Health Insurance Administration (NHI Administration)) decided to launch a pilot program (Signed review program) in 2016. Under the fee-for-service payment schemes for outpatient services and for the majority of inpatient services, the signed review policy was introduced along with a second-reviewer requirement for cases with deduction decisions, that is, the cases will be determined “deducted” only after two reviewers both agree on deduction decisions.

Originally, the NHI Administration conducted regular anonymous claims reviews performed by contracted medical doctors mainly working in teaching hospitals. Contracted senior reviewers routinely perform an appropriateness assessment of the reviews to assure their quality. These appropriateness reviewers are senior doctors with at least 20 years of practice experience and have served as NHI claims reviewers for at least 5 years. Starting in October 2016, the NHI signed review policy requested reviewers to disclose their identity to health care providers for cases with deductions. Yet, the appropriateness assessment remained anonymous because its purpose was to evaluate and ensure the reviewers’ implicit criteria for deduction decisions were at a similar level. The results of appropriateness assessment were valuable reference for reviewers’ reappointment, but were irrelevant to the deductions of reimbursement claims. This study aimed to evaluate the preliminary impact of the signed review policy on the reviewers’ decision regarding the reimbursement deduction rates and the appropriateness of the reviews.

## Results

### Characteristics of claim reviewers

Fifty-eight reviewers were included in the claims review process, with 1457 cases being reviewed in 2017, and these cases had been previously reviewed by 29 anonymous reviewers before the signed review policy in 2016. Table 1 shows the characteristics of the reviewers who were enrolled in this study. The majorities of the reviewers were male, were aged 46–59, and practiced at large-scale medical center hospitals. 48 (out of the 58) reviewers were included in the appropriateness assessment after the signed review policy with 695 reviewer reports. These cases had been previously reviewed anonymously by 27 reviewers, and their reviewer reports had also undergone an appropriateness assessment.

**Table 1:** Characteristics of the claims reviewers under the Taiwanese National Health Insurance scheme in this study.

Variables	Claims review				Appropriateness assessment			
	Anonymous review, 2016		Signed review, 2017		Anonymous review, 2016		Signed review, 2017	
	N	%	N	%	N	%	N	%
Total	29	100	58	100	27	100	48	100
Sex (male)	26	89.7	54	93.1	24	88.9	47	97.9
**Age**								
Young (= < 45)	2	6.9	21	36.2	2	7.4	19	39.6
Middle-aged (46–59)	17	58.6	29	50.0	17	63.0	23	47.9
Older (> = 60)	10	34.5	8	13.8	8	29.6	6	12.5
**Number of years of practice**								
High (> = 30)	11	37.9	9	15.5	10	37.0	7	14.6
Medium (21–29)	11	37.9	23	39.7	11	40.8	17	35.4
Low (= < 20)	7	24.1	26	44.8	6	22.2	24	50.0
**Hospital level**								
Medical center	17	58.6	35	60.3	15	55.6	28	58.3
Regional hospital	10	34.5	22	37.9	10	37.0	19	39.6
District hospital	2	6.9	1	1.7	2	7.4	1	2.1
Frequent reviewer (Yes)	16	55.2	39	67.2	16	59.3	32	66.7
**Specialty**								
Obstetrics/Gynecology	5	17.2	11	19.0	5	18.5	9	18.8
Urology	6	20.7	10	17.2	5	18.5	8	16.7
Otolaryngology	5	17.2	10	17.2	5	18.5	8	16.7
Ophthalmology	4	13.8	9	15.5	4	14.8	8	16.7
Neurology	3	10.3	6	10.3	2	7.4	4	8.3
Psychiatry	6	20.7	12	20.7	6	22.2	11	22.9

### Reimbursement deduction rates

Table 2 shows the reimbursement deduction rates under the anonymous review (2016) and signed review (2017) policies. When analyzed by cases (*N* = 1457), the overall deduction rate in 2016 was 33.56%, while the rate was 13.66% in 2017. When analyzed by orders (*N* = 12,237), the deduction rate was 5.35% in 2016, while the rate was 2.13% in 2017. Among the six specialties, all of the reductions in the deduction rate after the signed review policy reached the *P* < 0.01 significance level. The results from the GEE models (Table 3) revealed that after adjustments were made for potential confounding factors, the likelihood of deduction decisions for reimbursement cases decreased significantly after the signed review policy, with an odds ratio (OR) of 0.331 and a 95% confidence interval (CI) of 0.271–0.404 (*P* < 0.001). When using orders as the unit of analysis, we found similar results, with an OR of 0.392 and a 95% CI of 0.338–0.455 (*P* < 0.001). We also found that reviewers in obstetrics/gynecology, ophthalmology and neurology were less likely to make reduction decisions in case-based analysis. In the order-based analysis, female reviewers were more likely to make deduction decisions, while reviewers who worked in district hospitals, those who had been practicing for a small number of years and those who were in obstetrics/gynecology and urology were less likely to make deduction decisions.

**Table 2:** Deduction rate of the reimbursement claims before and after the signed review policy under the Taiwanese National Health Insurance scheme.

Variables	Deduction rate by cases			Deduction rate by orders		
	Number of cases	Anonymous review, 2016(%)	Signed review, 2017 (%)	Number of orders	Anonymous review, 2016 (%)	Signed review, 2017 (%)
**Specialty**						
Obstetrics/Gynecology	289	26.64	4.54	2037	5.06	0.79
Urology	243	30.45	20.58	2427	3.75	2.60
Otolaryngology	262	36.64	19.08	3096	4.10	1.49
Ophthalmology	226	36.28	11.50	1280	9.06	3.36
Neurology	146	28.08	4.11	1370	4.23	2.41
Psychiatry	291	40.89	18.21	2027	7.94	2.96
Total	1,457	33.56	13.66	12,237	5.35	2.13

All of the differences between 2016 and 2017 are statistically significant with *P* < 0.01.

**Table 3:** GEE models examining the effects of the signed review policy on the likelihood of deduction under the Taiwanese National Health Insurance scheme.

Variables	Deduction by cases (*N* = 1457*2)		Deduction by orders (*N* = 12,237*2)	
	OR	95% CI	OR	95% CI
Intercept	0.606*	0.403–0.910	0.116***	0.086–0.157
Signed review policy (Ref: anonymous review, 2016)	0.331***	0.271–0.404	0.392***	0.338–0.455
Sex (Ref: male)	1.549	0.994–2.415	2.406***	1.855–3.121
Inpatient cases (Ref: outpatient)	1.022	0.685–1.526	0.173***	0.137–0.219
**Hospital level (Ref: medical center)**				
Regional hospital	1.063	0.869–1.301	1.059	0.899–1.247
District hospital	0.764	0.397–1.469	0.272***	0.162–0.459
**Number of years of practice (Ref: high)**				
Medium	0.981	0.771–1.248	0.958	0.802–1.143
Low	0.868	0.609–1.238	0.681**	0.518–0.897
Frequent reviewer (Ref: no)	1.153	0.907–1.467	0.951	0.781–1.158
**Specialty (Ref: psychiatry)**				
Obstetrics/Gynecology	0.417***	0.294–0.592	0.695*	0.512–0.943
Urology	0.808	0.565–1.155	0.668**	0.513–0.869
Otolaryngology	0.933	0.681–1.278	0.997	0.783–1.270
Ophthalmology	0.706*	0.509–0.980	0.934	0.736–1.186
Neurology	0.459 ***	0.299–0.704	0.806	0.593–1.095

*P < 0.05, **P < 0.01, ***P < 0.001.

### Appropriateness assessment of review decision

**Figure 1:**
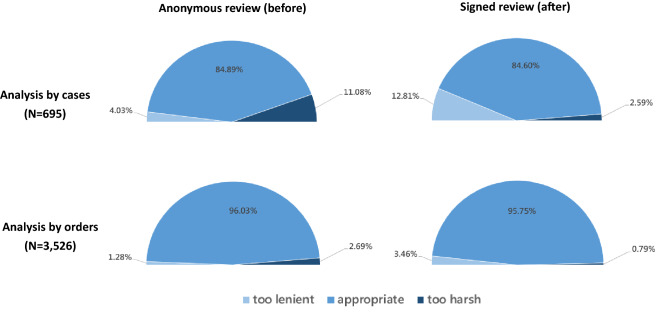
The distribution of inappropriate review decisions under anonymous and signed review.

Table 4 shows the results of the appropriateness assessment of the claims review decisions under the anonymous review (2016) and signed review (2017) policies. When analyzed by cases (*N* = 695), the overall rate of inappropriate decisions in 2016 was 15.11%, with 4.03% of them being “too lenient” and 11.08% being “too harsh”. In 2017, the rate was 15.40%, with 12.81% of them being “too lenient” and 2.59% being “too harsh”. When analyzed by orders (*N* = 3526), the overall rate of inappropriate decisions was 3.97%, with 1.28% of them being “too lenient” and 2.69% being “too harsh”. In 2017, the rate was 4.25%, with 3.46% of them being “too lenient” and 0.79% being “too harsh”. After the signed review policy, the percentage of the review decisions being “too lenient” increased significantly (*P* < 0.001), and the percentage of the decisions being “too harsh” decreased significantly (*P* < 0.001). When these rates were compared in the six specialty groups between 2016 and 2017, we found significant changes in four of the six groups. Figure 1 visualized the changes in “too lenient” and “too harsh” decisions before and after the signed review policy.

**Table 4:** Appropriateness assessment of the review decision under the Taiwanese National Health Insurance scheme.

Variables	Analysis by cases					Analysis by orders				
	Number of cases	Anonymous review, 2016 (%)		Signed review, 2017 (%)		Number of orders	Anonymous review, 2016 (%)		Signed review, 2017 (%)	
		Too lenient	Too harsh	Too lenient	Too harsh		Too lenient	Too harsh	Too lenient	Too harsh
**Specialty**										
Obstetrics/Gynecology	122	0.00	0.00	0.82	0.00	464	0.00	0.00	0.86	0.00
Urology	149	0.00	2.01	14.09***	0.00	737	0.00	0.68	2.58***	0.00
Otolaryngology	127	9.45	18.11	20.47*	3.94**	710	3.66	4.51	7.46**	1.13**
Ophthalmology	118	4.24	24.58	22.89***	7.63**	566	1.24	5.83	4.42**	1.59**
Neurology	56	7.14	1.78	8.93	1.78	336	1.19	0.30	1.49	0.89
Psychiatry	123	5.69	17.07	7.32	2.44**	713	1.12	3.37	2.24	0.84**
Total	695	4.03	11.08	12.81***	2.59***	3526	1.28	2.69	3.46***	0.79***

*P < 0.05, **P < 0.01, ***P < 0.001 when comparing the percentages of “too lenient” and “too harsh” between 2016 and 2017.

Finally, the results from the multinomial logistic regression using GEE models (Table 5) show that the inappropriate review decision was more likely to be “too lenient” (than to be appropriate) after the signed review policy, with an OR of 2.657 (95% CI = 1.582–4.463, *P* < 0.001) analyzed by cases or an OR of 3.333 (95% CI = 2.288–4.855, *P* < 0.001) analyzed by orders. On the other hand, the inappropriate review decision was less likely to be “too harsh” (than to be appropriate) after the signed review policy, with an OR of 0.382 (95% CI = 0.203–0.716, *P* < 0.01) analyzed by cases or an OR of 0.345 (95% CI = 0.197–0.603, *P* < 0.001) analyzed by orders. In the analysis by cases, we also found that reviewers with a low or medium number of years of practice (compared with those with a high number of years of practice) were more likely to make inappropriate decisions that were considered “too lenient”. Variations existed among the six specialty groups; for example, in models analyzed by both case and order, specialists in otolaryngology were more likely to make decisions that were “too lenient”, while specialists in urology were less likely to make decisions that were “too harsh”.

**Table 5:** Multinomial logistic regression of GEE models examining the effect of the signed review policy on the appropriateness of the review decision under the Taiwanese National Health Insurance scheme.

Variables	Analysis by cases (*N* = 695*2)				Analysis by orders (*N* = 3526*2)			
	Too lenient (Ref: reasonable)		Too harsh (Ref: reasonable)		Too lenient (Ref: reasonable)		Too harsh (Ref: reasonable)	
	OR	95% CI	OR	95% CI	OR	95% CI	OR	95% CI
Intercept	0.004		0.291		0.022***		0.006***	
Signed review policy (Ref: anonymous review, 2016)	2.657***	1.582–4.463	0.382**	0.203–0.716	3.333***	2.288–4.855	0.345***	0.197–0.603
Sex (Ref: male)	5.758**	1.916–17.303	1.501	0.235–9.581	0.446	0.138–1.442	1.341	0.328–5.482
**Hospital level (Ref: district hospital)**								
Medical center	4.951	0.901–27.224	1.007	0.049–20.727	0.291*	0.096–0.881	4.400	0.314–61.569
Regional hospital	4.022	0.708–22.842	0.594	0.027–13.14	0.269*	0.087–0.831	2.797	0.190–41.108
**Number of years of practice (Ref: high)**								
Medium	2.379**	1.260–4.491	0.587	0.329–1.048	1.205	0.813–1.787	0.791	0.509–1.227
Low	2.693**	1.336–5.429	0.144*	0.03–0.684	0.556*	0.331–0.934	0.826	0.354–1.93
Frequent reviewer (Ref: yes)	1.384	0.875–2.191	1.189	0.615–2.299	1.259	0.861–1.841	2.141*	1.027–4.464
**Specialty (Ref: psychiatry)**								
Obstetrics/Gynecology	0.041**	0.005–0.319	< 0.001	< 0.001– > 999.999	0.287*	0.098–0.840	< 0.001	< 0.001– > 999.999
Urology	1.108	0.541–2.270	0.081***	0.023–0.289	0.909	0.483–1.712	0.275**	0.114–0.663
Otolaryngology	2.679**	1.376–5.218	0.910	0.458–1.809	3.861**	2.321–6.422	1.162	0.688–1.962
Ophthalmology	1.626	0.811–3.260	1.983*	1.108–3.549	1.992*	1.122–3.535	1.631*	1.000–2.660
Neurology	0.995	0.359–2.756	0.272	0.046–1.589	1.179	0.501–2.778	0.484	0.15–1.566

*P < 0.05, **P < 0.01, ***P < 0.001.

## Discussion

This study examined the effect of removing anonymity protection on the deduction decisions in health insurance claims review. We found that the signed review policy significantly reduced the reimbursement deduction rate under the Taiwanese NHI scheme. The deduction rate greatly decreased by approximately 67% at the case level (OR = 0.331) or 61% at the order level (OR = 0.392). In terms of the appropriateness of the claims review decisions, the signed review policy slightly increased the proportion of inappropriate review decisions from 15.11% to 15.40% at the case level and from 3.97% to 4.25% at the order level. However, the type of inappropriate decision changed dramatically at both the case and order levels, with a dramatic increase in decisions that were “too lenient” (OR = 2.657 and 3.333, respectively) and a significant decrease in decisions that were “too harsh” (OR = 0.382 and 0.345, respectively) after the signed review policy.

Findings from this study revealed that anonymity protection played a major role in claims deduction decisions. Reviewers were less likely to make deduction decisions when their identity would be disclosed than were reviewers under anonymity protection. The NHI signed review policy disclosed the reviewer’s identity in the claims review report, which led to a 61–67% decrease in claims deduction rates. The findings also show significant variation in the deduction rates among the six specialties especially after the signed review policy. Since the deduction decision was made by the reviewer mainly based on the clinical guidelines, a lower deduction rate might represent a higher level of compliance with the clinical practice guidelines.

Our finding is similar to those of some reports in the “open review” of the journal article review process^17^, although some only show very little effect^18^. In addition, under the open review policy, fewer reviewers are willing to perform external review for academic journals^16,19^. Similarly, except for the six specialties in this study, no other specialty societies thus far have been willing to participate in the signed review program. An internal study of the NHI Administration showed that the concerns of peer pressure, offending senior physicians and receiving public criticism were the main reasons that discouraged the reviewers’ willingness to participate in the signed review program.

Regarding the appropriateness of the claims review decisions, we also found significant changes after the signed review policy under the Taiwanese NHI scheme. Among those assessed to be inappropriate review decisions, the signed review policy led to a dramatic increase in decisions that were “too lenient” and a large decrease in decisions that were “too harsh” when compared with the decisions made under anonymity protection. The findings reveal that removing anonymity protection might have changed the intrinsic standards of the reviewers. However, the findings may also be due to the poor reliability of peer review of quality of care, which has been discussed for a long time^8,20–23^. In our study, we used the same set of claims cases to compare the reviewer’s decisions with and without anonymity protection, which may have decreased the reliability problem to some extent. In addition, it has been reported that in the US, PROs have been considered agents of cost containment rather than quality assurance^1^. Our findings show that adopting a signed review policy greatly reduced the deduction rate of reimbursement claims under the NHI single-payer system in Taiwan. However, we are unable to judge whether the deduction rate is reasonable or not under the signed review policy.

It should be noticed that the result may be possibly influenced by the requirement of the “second review” for deducted cases under signed review policy. To minimize its potential influence, this study has excluded the review decisions of the second reviewer, that means, the review decisions in this study were made by the first reviewer only. In addition, a NHIA internal study showed that identity disclosure was the main concern for physicians when they were making choice to be a reviewer or not. Therefore, it is reasonable to infer that the impact of second review on the results might be limited, and the decrease in the deduction rate is mainly attributed to “signed review” policy, rather than” second review” requirement.

The limitations of this study should be mentioned. Although we selected the cases and assigned them to the reviewers randomly, the participating reviewers might not be representative of claims reviewers in general. Due to very small numbers in several subgroups in the appropriateness analysis, the coefficients of estimation in the GEE model seemed unstable. Due to the low level of willingness of physicians to participate in signed review, the findings might not represent the overall changes in the six specialties. Finally, there are unique features of the compulsory NHI system in Taiwan that may limit the generalizability of our findings to other health systems, for example, patients possess the freedom to choose a preferred physician for each visit at a community clinic or the outpatient department in a hospital without referral at a low copayment level^24^.

In summary, this real-world study revealed that removing anonymity protection for the claim reviewers changed their review decisions significantly. A signed review policy under a universal health scheme in Taiwan dramatically reduced the claims deduction rate and increased the tendency of decisions that were “too lenient”. There might be no best way to perform utilization reviews in health care services; maintaining anonymity or not is a decisional challenge for health authorities and health insurers around the globe.

## Methods

### Reviewers selection under the signed review policy

During the introduction period of the signed review policy, the NHI Administration asked medical specialist societies to recommend potential claims reviewers who were willing to disclose their identity collectively in the NHI quarterly review report. In October 2016, six specialist societies voluntarily agreed to participate in the signed review program: obstetrics/gynecology, urology, otolaryngology, ophthalmology, neurology, and psychiatry. These reviewers who had actively conducted anonymous utilization reviews in 2016 for the NHI Administration and who had subsequently joined the signed review program started in October 2016 were randomly selected in the study. A total of 58 physicians who frequently or regularly attended utilization review were enrolled.

### The claim cases and re-review process

The study was conducted in 2017 at the Taipei branch of the NHI Administration, which is the largest branch of the six branches and accounts for approximately one-third of the reimbursement claims nationwide.

The reimbursement claim records of outpatient and inpatient services were retrieved from January to June 2016, which had been reviewed anonymously and reimbursed. These claim records were then randomly and proportionally selected according to the six specialties and the number of participating reviewers (approximately 25–30 cases per reviewer). These claim records were randomly assigned to each of the reviewers via a regular electronic review platform during July and October 2017. All the claims reviewers were not aware of this study, and they considered the claims records to be regular cases under the signed review policy.

Once the reviewer considered that certain orders tended to be “not medically necessary “, these orders and the amount of money incurred by them would be deducted from the claimed amounts. With second review requirement under signed review policy, the review results could be distinguished into two parts, including the review decisions of first reviewers and the re-review deduction decisions of second reviewers. The study only included the review decisions of first reviewers for subsequent analysis. In this study, the deduction rate was calculated in two different units of analysis, i.e., by cases and by orders.

### Appropriateness evaluation of reviewer decisions

After the claims reviewers completed the review process for the study cases, these review reports were randomly selected with 15–20 cases for each reviewer for an appropriateness assessment. In the study, the claims reviewers and the appropriateness assessors were two group of physicians. Several appropriateness assessors have participated in the claims review; those cases reviewed by them were deleted from the list for appropriateness assessment. Therefore, all the claims review cases included in this study were conducted by one set of reviewers and underwent appropriateness assessment by another set of senior reviewers. In addition, when a claims reviewer was working in the same hospital with an appropriateness assessor, his/her review cases were also excluded from the list for appropriateness assessment. Therefore, all appropriateness assessors would not perform assessment on their own cases nor the cases from their own hospitals. By the way, the appropriate assessors were unable to know the identities of the claims reviewers of the reports they assessed. Among the 58 claims reviewers, 10 of them and their cases were deleted from the list for appropriateness assessment. The appropriateness assessment consisted of three results categories: “appropriate”, “too lenient” or “too harsh” which were reported by the assessors.

### Statistical analysis

Generalized estimation equations (GEEs) were employed to estimate the effects of the signed review policy on the deduction decision and appropriateness assessment while taking the feature of before-and-after study design into account and controlling for potential confounders. This study incorporated potential confounders in the analysis, including sex, age, specialty, year of practice, hospital level, frequent reviewer and cases type (inpatient/outpatient). The reviewers’ ages were not included in the regression models due to collinearity concerns with the reviewers’ number of years of practice.

## Data Availability

Raw data of the study were retrieved from National Health Insurance electronic claims dataset and the reviewer’s decision reports. The original dataset used in the study is not publicly available. The data supporting the finding and analysis of the study are however available upon request with approval of the National Health Insurance Administration.
